# Measurement of local temperature increments induced by cultured HepG2 cells with micro-thermocouples in a thermally stabilized system

**DOI:** 10.1038/s41598-017-01891-1

**Published:** 2017-05-11

**Authors:** Fan Yang, Gang Li, Jiamin Yang, Zhenhai Wang, Danhong Han, Fengjie Zheng, Shengyong Xu

**Affiliations:** 1 Key Laboratory for the Physics & Chemistry of Nanodevices, and Department of Electronics, Peking University, Beijing, 100871 P. R. China; 2 School of Basic Medical Sciences, Beijing University of Chinese Medicine, Beijing, 100871 P. R. China

## Abstract

To monitor the temperature distribution of a cell and its changes under varied conditions is currently a technical challenge. A variety of non-contact methods used for measuring cellular temperature have been developed, where changes of local temperature at cell-level and sub-cell-level are indirectly calculated through the changes in intensity, band-shape, bandwidth, lifetime or polarization anisotropy of the fluorescence spectra recorded from the nano-sized fluorescent materials pre-injected into the target cell. Unfortunately, the optical properties of the fluorescent nano-materials may be affected by complicated intracellular environment, leading to unexpected measurement errors and controversial arguments. Here, we attempted to offer an alternative approach for measuring the absolute increments of local temperature in micro-Testing Zones induced by live cells. In this method, built-in high-performance micro-thermocouple arrays and double-stabilized system with a stability of 10 mK were applied. Increments of local temperature close to adherent human hepatoblastoma (HepG2) cells were continuously recorded for days without stimulus, showing frequent fluctuations within 60 mK and a maximum increment by 285 mK. This method may open a door for real-time recording of the absolute local temperature increments of individual cells, therefore offering valuable information for cell biology and clinical therapy in the field of cancer research.

## Introduction

Temperature is an important physical parameter in organisms. A great number of biological activities occurring in cells, such as enzyme reaction^1^ and metabolism^2^, are found accompanied by temperature increments or fluctuations^3, 4^. Accurate measurement of the local temperature variation of individual cells and the intracellular temperature distribution may offer valuable clues for understanding the mechanism of heat generation and heat diffusion in different organelles, and therefore promote the development of research on the pathogenesis of cancer and other diseases^5–8^. However, a reliable method for precise measurement of local cellular temperatures remains a technical challenge to date.

Over the past decade, researchers have made great efforts to explore various techniques for the measurement of intracellular temperature^9–12^. From the sensing mechanism, these techniques may be divided into two categories. One is using thermal sensitive fluorescent materials for non-contact measurements, the other is using contact thermometers to measure the cellular temperature. In the non-contact luminescent methods, measurement of temperature is based on the thermo-sensitive physical properties^13^ of fluorescent materials that changed with temperature variations, for examples, intensity of fluorescence^14, 15^, band-shape of fluorescence^5^, bandwidth of fluorescence^16^, fluorescence lifetime^17^ and fluorescence polarization anisotropy^18^. The thermo-sensitive fluorescent materials applied for luminescent measurements include nanoparticles^19^, nanodiamonds^20^, nanogels^15^, quantum dots^21, 22^, fluorescent copolymers^23, 24^, green fluorescent proteins^25, 26^, and etc. For example, Okabe *et al*.^17^ introduced a hydrophilic fluorescent polymeric thermometer to investigate the temperature distribution within COS-7 live cells. They observed that the nucleus could have a 0.96 K higher temperature than the cytoplasm. Interestingly, the temperature gap between nucleus and cytoplasm differed depending on various status of the cell cycle. As fluorescent materials could be made into nano-size, these non-contact cellular temperature measurement methods can provide very good spatial resolution. By using small molecule fluorescent thermometers, Itoh *et al*.^27^ measured heat production in single myotubes after Ca^2+^ burst. Using similar method, Arai *et al*. monitored the intracellular temperature changes at the mitochondria^27^ and endo/sarcoplasmic reticulum^28, 29^ in different cell lines. Unfortunately, the properties of some fluorescent materials injected into live cells by cellular endocytosis or electroporation technique might be affected by the ion concentration, micro-viscosity, Ca^2+^ ion concentration and pH value in the complex intracellular fluid environment^30^, and it needs complicated calibration process to obtain the actual measured temperature value, therefore leading to a temperature resolution ranging between 0.1–0.5 K^11, 17, 31, 32^. In rare cases, Uchiyama *et al*.^33^ reported that a temperature resolution could be as high as 0.01 K by using cationic fluorescent polymeric thermometers.

Recently, intracellular temperature fluctuation and distribution have attracted much attention. Indeed, the validity of subcellular temperature changes reported in various papers has been challenged^34, 35^. Baffou *et al*.^34^ estimated the intracellular temperature variation should be about 10^−5^ K, rather than the reported 0.5–2 K in previous experiments. Lacking of solid experimental data, the exact variation of cellular temperature in individual live cells remains as a controversial issue so far^36, 37^.

The above argument might be ceased if reliable and accurate measurement data for the local temperature of individual cells could be obtained. To avoid the relatively large measurement error in non-contact measurements, contact methods using miniaturized probes are recommended as a good alternative. Suzuki *et al*.^38^ developed a microthermometer by filling a glass micropipette with thermosensitive fluorescent dye Europium (III) (thenoyltrifluoroacetonate trihydrateare) to measure the inner temperature of a single cell. Small thermocouples based on the Seebeck effect^39^ were also developed for such single-cell measurement. Because of their passive nature, thermocouples at the microscale and even nanoscale^40^ could offer reliable and highly accurate data in local temperature measurements^41^. Watanabe *et al*.^42^ first used glass micropipettes with 1 μm diameter to develop platinum/gold (Pt/Au) micro-thermocouple probes for intracellular temperature measurement. Wang, C. *et al*.^43, 44^ observed various single-cell temperature fluctuations under different stimulus with nano-tungsten/platinum (W/Pt) thermocouple probes. These probes were fabricated with a sandwich structure and had a thermopower ranging between 6–8 μV/K. The temperature resolution of these probes was 0.1 K. Compared with luminescent thermometers, thermocouple probes have a relatively larger size, but they offer much more accurate temperature resolution. Moreover, the measurement system applied for thermocouple thermometers is usually much less costly than that for the luminescent methods. However, the temperature resolution of the reported data with contact methods is in the range of 100 mK, and it needs to be improved to the level of 1–10 mK as achieved in molecular devices^33^.

To counter the problems mentioned above, a solution for this challenge is presented in this paper. A specially designed thermally double-stabilized constant-temperature system was set up for measuring the increments of local temperature in Testing Zones which were used for culturing target cells. By using highly sensitive Pd/Cr (and Cr/Pt) micro-thermocouple arrays with a thermopower of 20.99 ± 0.1 μV/K (and 15.59 ± 0.3 μV/K) and putting all the major measurement instruments into the double-stabilized constant-temperature system, a reliable stability of ±5 mK was achieved for the measurement system. With this highly stable system, weak increments of local temperature in Testing Zones induced by the cellular activities of cultured adherent HepG2 cells were detected. Without additional chemical or mechanical stimulus, the maximum recorded increment of local temperature in Testing Zone was 285 mK. While in most cases, recorded temperature increments were less than 60 mK. In this work, we attempted to record the temperature variation at cell’s membrane without stimulus. This may shed light on the accurate measurement of the temperature fluctuations of individual cells by using the miniaturized passive thermocouples in a stable measurement system.

## Results and Discussions

### Performance of micro-TFTC arrays

**Figure 1:**
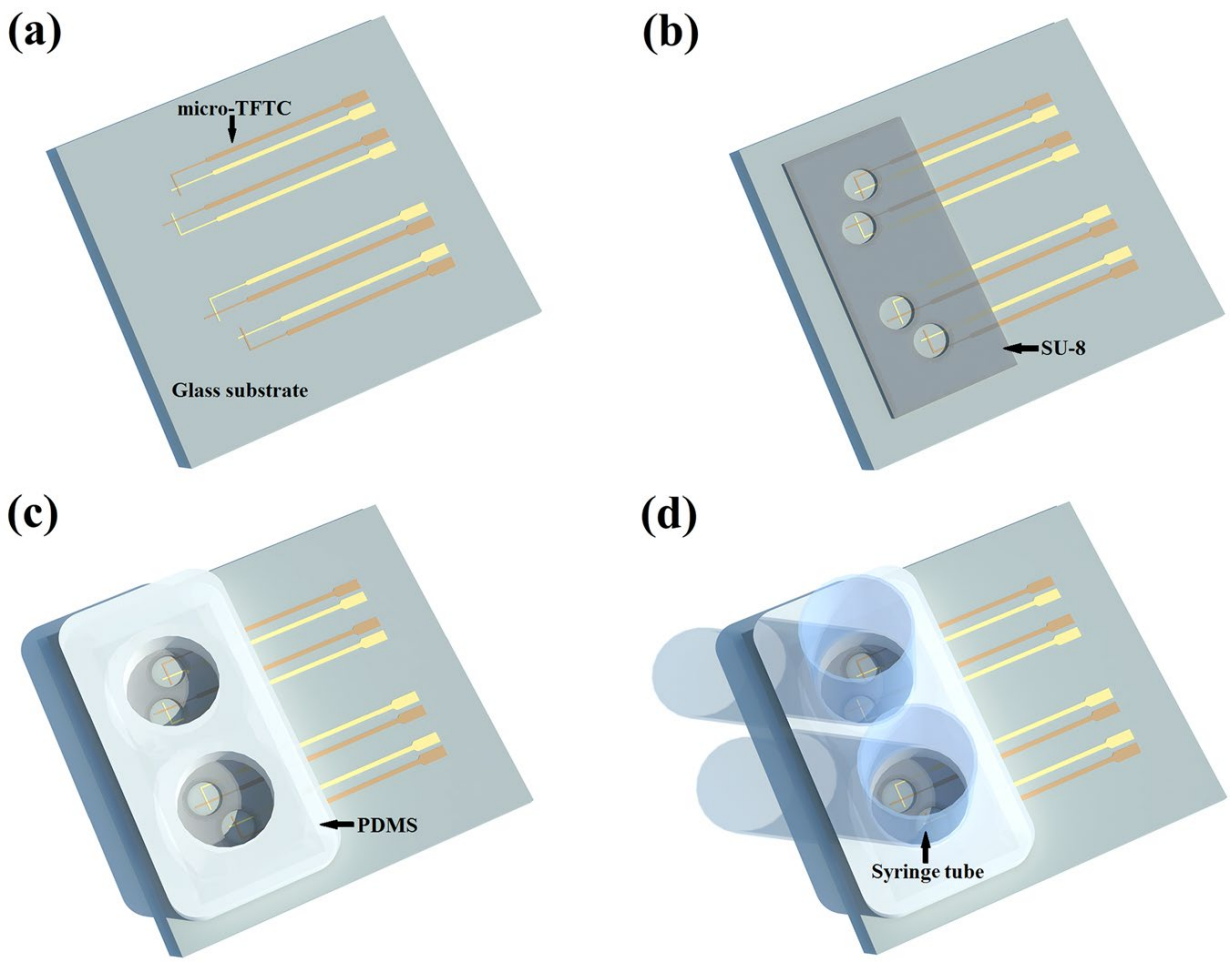
An illustration for the 3D structures of a testing device. (**a**) A micro-thin-film thermocouple (TFTC) array is fabricated on the glass substrate. (**b**) Testing Zones are confined by a layer of SU-8 above the micro-TFTC array. (**c**) Cylindroid rooms are confined by a layer of PDMS above the Testing Zones. (**d**) Syringe tubes are mounted to the PDMS layer at the holes’ positions.

Figure 1 illustrates three dimensional (3D) structures of the key elements of testing device. On a glass substrate, the bottom layer of the testing device is a micro-thin-film thermocouple (TFTC) array made with Pd/Cr or Cr/Pt thin-film stripes (Fig. 1(a)). Then an SU-8 layer is applied to confine shallow circular wells (Fig. 1(b)), which are defined as “Testing Zones”. The Testing Zone is the place where the target adherent cells are supposed to stick firmly to the micro-TFTCs surface. The thermal capacitance for an individual micro-TFTC, with a film thickness of 50–100 nm and a stripe width of 2–3 μm, is calculated to be 8.0 × 10^−13^–1.6 × 10^−12^ J/K per micron length, as the thermal capacitance *C* can be obtained by *C = cρV*, where *c* is the specific heat capacity of the metallic thin-film stripe(s), *ρ* is the material density, and *V* is the effective volume of the sensor. For the Pd, Cr, and Pt metallic thin-film stripe(s), their specific heat capacity are 240 J/Kg·K, 450 J/Kg·K and 130 J/Kg·K, and their material density are 12.02 g/cm^3^, 7.19 g/cm^3^ and 21.45 g/cm^3^. In this work, an effective length of 20 microns is taken for a TFTC which takes into account two metal thin-film stripes of 12 microns in length as well as two metal thin-film disks of 8 microns in diameter. The effective thermal capacitance of this piece of TFTC is calculated to be 3 × 10^−11^–6 × 10^−11^ J/K. For a single adherent HepG2 cell (roughly 15–25 μm in diameter), it is approximately simplified as a water ball with a diameter of 20 microns. By using the specific heat capacity of water of 4.2 × 10^3^ J/Kg·K and a density of 1.0 g/cm^3^, a thermal capacitance of 1.76 × 10^−8^ J/K is obtained. This value is about 300–600 times larger than that of a TFTC, so the micro-TFTC sensors at the Testing Zone will serve well as thermal sensors for the measurement of temperature increments induced by target cells. Next, a PDMS layer roughly 10 mm in thick is used to define large cylindroid rooms (7 mm in diameter) for containing the culture medium (Fig. 1(c)). Finally, syringe tubes of 2.5 mL are mounted to the PDMS layer at the holes’ positions (Fig. 1(d)) for expanding the volume of PDMS cylindroid rooms, so that 3 mL culture medium can be filled for a continuous culturing process of tens of hours each time. Enough nutritional supply is critical for this work. Because the cells usually randomly distribute on the substrate surface, it is not certain that at least one cell goes to the micro-TFTC position and firmly sticks to the micro-TFTC surface after one fill of adherent cells into the testing device. Therefore, a reasonable expectation is that after the cells are cultured for one or a few life cycles, the adherent cells will gradually cover all the bottom surface of the 7-mm diameter cylindroid room made by PDMS layer, including the micro-TFTC array area. Then increment of local temperature in Testing Zone will be detected if the increment is larger than the sensitivity of the micro-TFTCs. So, enough nutritional supply can ensure that all the measurements are performed without any interruption for several life cycles of the cultured cells. Photographs of fabrication processes for the device with build-in micro-TFTC array are shown in Fig. S1 of Supplementary Information.

**Figure 2:**
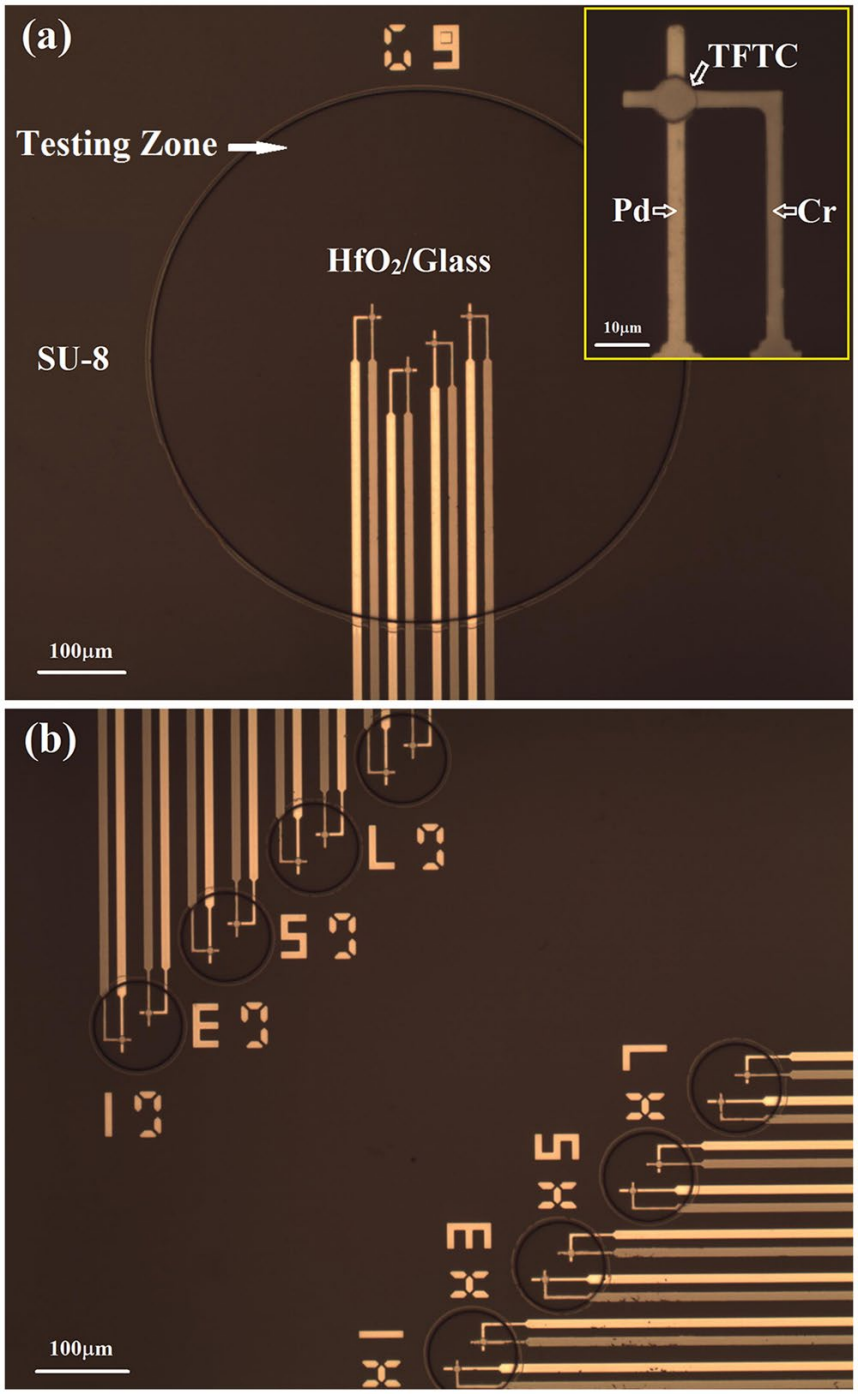
Optical photographs of different Testing Zones. (**a**) A bigger Testing Zone with a diameter of 600 μm. It contains 4 Pd/Cr micro-TFTCs. The inset presents one of the Pd/Cr micro-TFTCs for details. The darker stripe is Cr stripe, the brighter stripe is Pd stripe. The stripe width is 3 μm. The overlapping junction on the top is the hot-end of the micro-TFTC. It has a diameter of 8 μm. (**b**) Two arrays of smaller Testing Zone with a diameter of 100 μm. Each Testing Zone contains 2 Pd/Cr micro-TFTCs.

Figure 2 presents optical photographs of two types of the Testing Zone with different diameters. The bigger one has a diameter of 600 μm, containing 4 Pd/Cr micro-TFTCs (Fig. 2(a)). On the top of these sensors, there is a 2-nm thick insulating layer of HfO_2_. Leaking test showed that the corresponding resistance between two neighboring TFTC sensors in the same Testing Zone in the HCl aqueous solution with pH = 2 was 10^8–9^ Ohm. This gives sufficient electrical isolation for the micro-TFTCs to detect weak signals in culture medium, whose pH value started at near 7 as fresh medium and ended around 6 after culturing cells for tens of hours. The insulating layer ensured that the output readings of TFTC sensors were independent of the variations of pH value and ion concentration in the testing environment (see Fig. S2 in Supplementary Information). One of the Pd/Cr micro-TFTCs is shown in the inset for details, whose stripe width is 3 μm. The circular overlapping junction of these two metallic stripes on the top is the temperature sensing area (this is also the “hot end”) of the micro-TFTC, which is 8 μm in diameter. The Cr stripe appears darker than the Pd stripe. In the bottom panel (Fig. 2(b)), it shows two arrays of smaller Testing Zone with a diameter of 100 μm, and each Testing Zone contains 2 TFTCs of the same type shown in Fig. 2(a).

**Figure 3:**
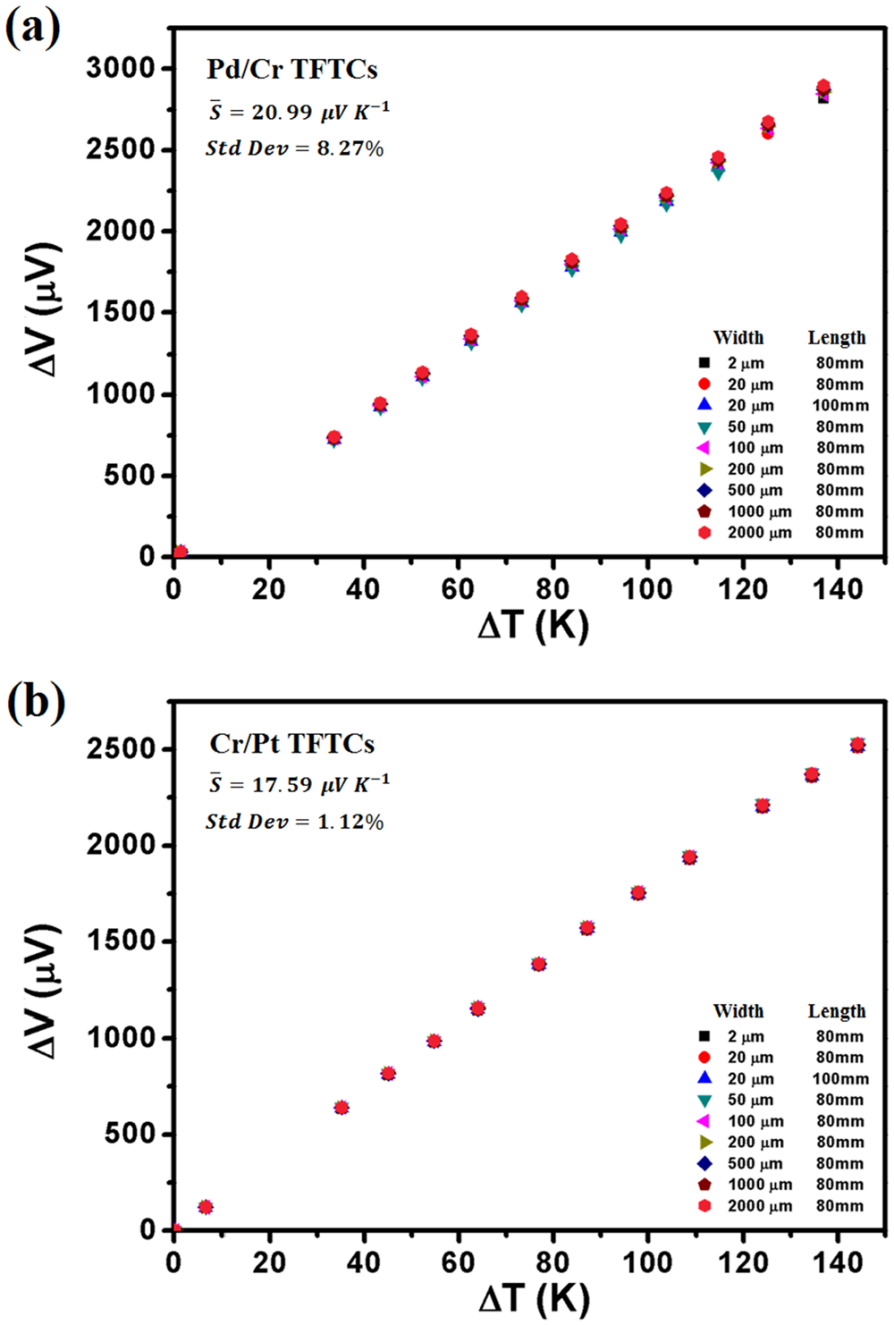
Calibration results of the Pd/Cr and Cr/Pt TFTCs. The calculated thermopower is the average value of 9 different TFTCs. (**a**) Calibration results of 9 different Pd/Cr TFTCs. These TFTCs have varied stripe width of 2 μm, 20 μm, 50 μm, 100 μm, 200 μm, 500 μm, 1000 μm, and 2000 μm, and different stripe length of 80 mm and 100 mm. The calculated thermopower of Pd/Cr TFTCs is 20.99 ± 0.1 μV/K, with a standard deviation of 8.27%. (**b**) Calibration results of 9 different Cr/Pt TFTCs. These TFTCs have the same size with the Pd/Cr TFTCs. The calculated thermopower of Pd/Cr TFTCs is 17.59 ± 0.3 μV/K, with a standard deviation of 1.12%.

Figure 3 plots the calibration results of the Pd/Cr and Cr/Pt TFTCs. The calibration results for a bunch of Pd/Cr TFTCs, which shared the same thickness of 50 nm for Pd stripes and 80 nm for Cr stripes, are presented in Fig. 3(a). These Pd/Cr TFTCs were designed with varied stripe width ranging between 2 μm and 2000 μm and different stripe length of 80 mm or 100 mm. From Fig. 3(a), it can be seen that different TFTCs share the same linear relationship between the output potential difference and temperature difference. It shows that the thermopower of these TFTCs is insensitive to the stripe width and length, which is consistent with previous research conclusion^45^. The calculated thermopower of Pd/Cr TFTCs is 20.99 ± 0.1 μV/K. It is an average value of 9 different TFTCs with a standard deviation of 8.27%. The calibration data of these sensors are listed in Table S1 of Supplementary Information. In some testing devices, Cr/Pt micro-TFTCs with a thermopower of 15.59 ± 0.3 μV/K were applied. The calibration results and calibration data of Cr/Pt TFTCs are plotted in Fig. 3(b) and listed in Table S1 of Supplementary Information, respectively.

### Stability of the cellular temperature measurement system

**Figure 4:**
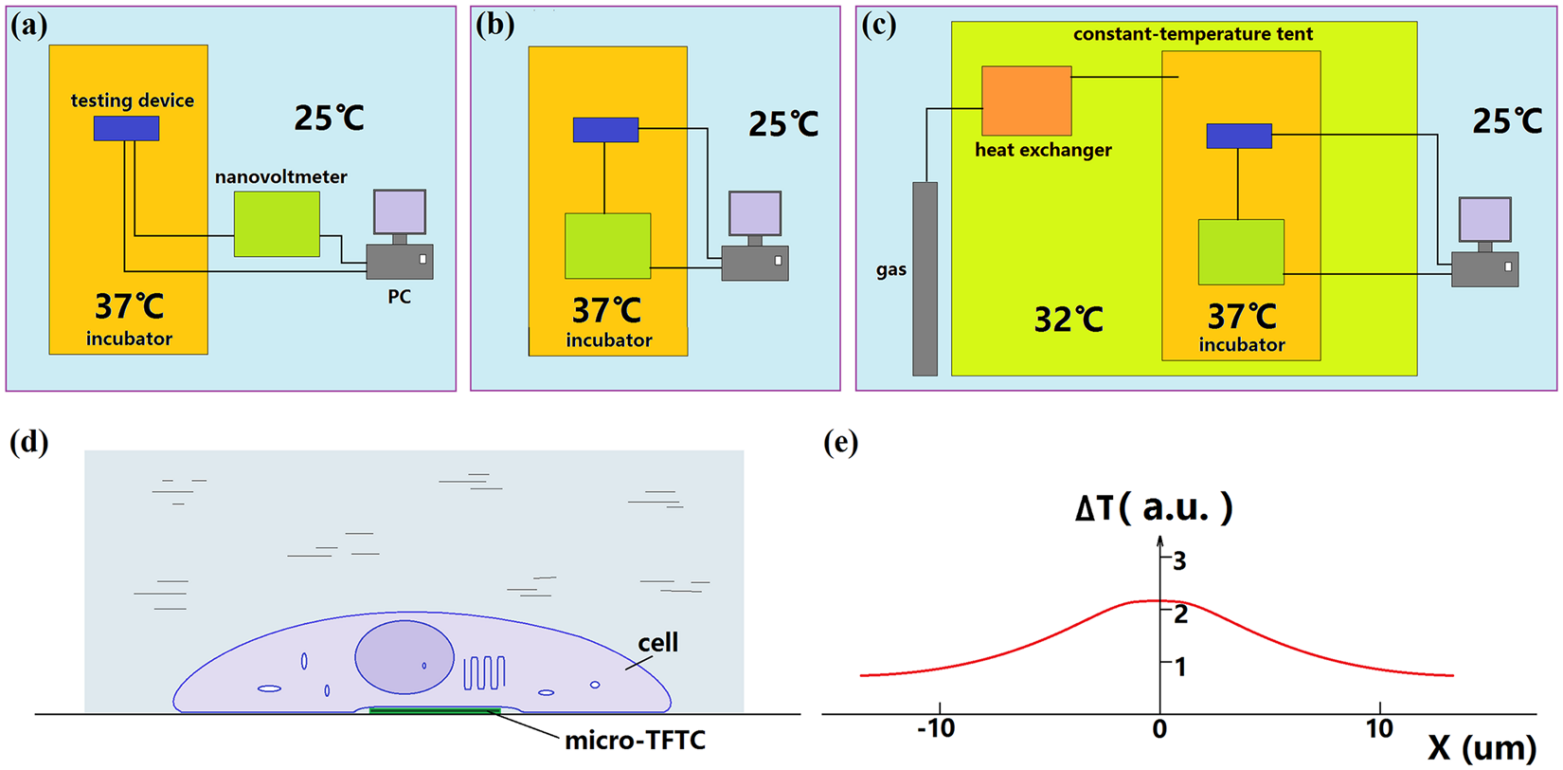
**S**chematic of the structures of measurement system and the measurement of local cellular temperature. (**a**) In normal practice, the testing device is put into the incubator at 37 °C and the other measurement instruments are put on the outside of the incubator at 25 °C. (**b**) The testing device and the other measurement instruments are put into the incubator at 37 °C, leaving the computer and monitor outside of the incubator at 25 °C. (**c**) A big constant-temperature tent kept at 32 °C is put on the outside of the incubator. Testing device and measurement instruments are put inside the incubator. The CO_2_ gas tank, computer and monitor are put on the outside of the tent. (**d**) A live cell firmly sticks to the micro-TFTC sensor. (**e**) A non-uniform temperature distribution in the cell (15–25 μm in diameter).

In this work, a special attention was paid to the setup of thermally double-stabilized constant-temperature measurement system. In normal practice, the testing device with built-in micro-TFTCs is put into a constant-temperature incubator where cells are cultured, and the other measurement instruments are put on the outside of incubator and connected to the testing device by lead cables as schematically illustrated in Fig. 4(a). During the cell culturing process, a temperature gradient around 12 K (e.g., 37 °C for cell culturing and 25 °C as room temperature) is often established between the temperature of cells under test and the cold-ends of sensor leads. This setting with a temperature gradient of 12 K can bring large thermal noise. In addition, ideally the lead cables connected to the TFTCs should be the same materials as that of the Pd/Cr (or Cr/Pt) micro-TFTCs to eliminate additional error. However, for an array with 96 micro-TFTCs, this same-material-lead strategy may be simply too difficult to accomplish. On the other hand, even the lead problem can be solved, keeping the cold-ends of the sensor leads at a constant temperature with a stability better than 100 mK is a difficult task. To address these issues, a wise practice is to put the testing device with built-in micro-TFTC array, the multiplexer, the Keithley 2182 A nanovoltmeter, as well as all the connecting cables into the same commercial constant-temperature incubator, whose thermal stability is around 0.1 K, as shown in Fig. 4(b). This setting can reduce the thermal noise effectively if the incubator could run stably. According to the thermodynamic principle and the instructions of this incubator, it will work more stable at an environment temperature 5 °C lower than the preset temperature. Hence, a controllable home-designed constant-temperature tent is set up for this commercial incubator as schematically illustrated in Fig. 4(c). The stability of this home-designed tent is around 0.5 K when working in a laboratory where an air-conditioner is kept running. In addition, although the CO_2_ gas cylinder needed for cell culture is put on the outside of the constant-temperature tent for security consideration, the CO_2_ gas can be warmed up through a long, curved pipe mounted inside the tent before it is introduced into the incubator. The inverted microscope used in the measurement is also put into the tent. Only computer and monitor of the system are located on the outside of the constant-temperature tent. Photographs of this thermally double-stabilized constant-temperature cellular temperature measurement system are shown in Fig. S3 of Supplementary Information.

**Figure 5:**
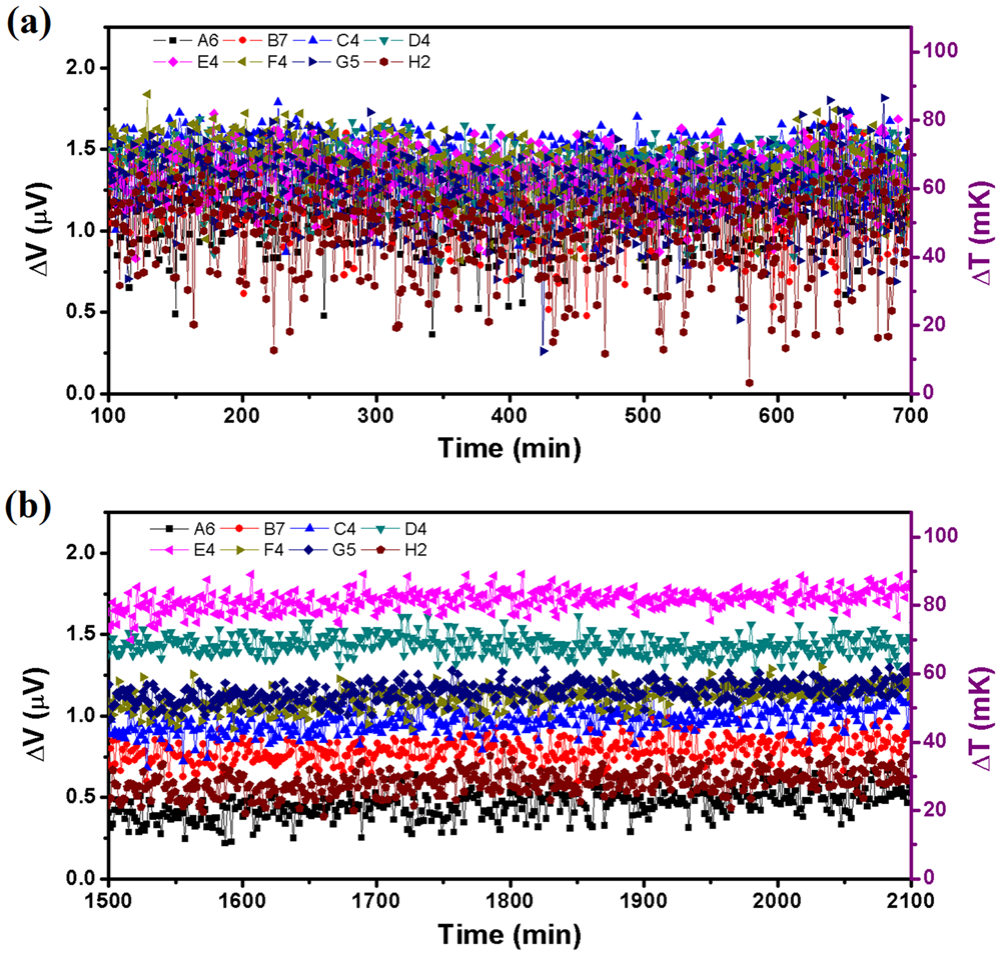
Thermal fluctuations of 8 Pd/Cr micro-TFTCs under different conditions. (**a**) Thermal fluctuations of 8 Pd/Cr micro-TFTCs (A6, B7, C4, D4, E4, F4, G5 and H2 located in different Testing Zones of different regions) tested in an ordinary laboratory kept at 25 °C, which were exposed to the air. (**b**) Thermal fluctuations of the same TFTCs tested in the double-stabilized constant-temperature system kept at 32 °C, which were covered by culture medium.

With these unique system parameters, a thermal stability of 10 mK was achieved. Figure 5 presents a clear comparison for the difference in thermal fluctuations recorded by the same testing device with a Pd/Cr micro-TFTC array, between the ones exposed to the air and located in an ordinary laboratory at room temperature of 25 °C (Fig. 5(a)), and those covered by culture medium and located in the double-stabilized constant-temperature system kept at 32 °C (Fig. 5(b)). In both cases, the whole micro-TFTC array (including hot-ends and cold-ends), the leads and the measurement instruments were all kept at the same temperature. Theoretically, the output readings of the micro-TFTC array should be zero. Yet, temperature fluctuation and electrical noise in the measurement system existed in both cases, therefore non-zero data have been recorded. From Fig. 5(a), it can be seen that, for the device and instruments located in an ordinary laboratory, the average thermal fluctuation is ±0.5 μV, corresponds to ±24 mK. While in Fig. 5(b), for the testing device and instruments located in the double-stabilized constant-temperature system, the average thermal fluctuation is only ±0.1 μV, or ±5 mK, which is improved by 4.8 times. From this comparison, it can be regarded that a double-stabilized constant-temperature measurement system with the thermal stability of 10 mK has been set up.

In order to set blank control group for the measurement, each of the testing device was divided into four regions. And in each region, there are 24 micro-TFTCs distributed in the two big (diameter 200 μm, 400 μm, 600 μm or 800 μm, corresponding to different regions) and eight small (diameter 50 μm or 100 μm) Testing Zones. For calibration purpose, control experiments for each testing device before and after the cellular temperature measurement were performed. Three of the four regions were filled with culture medium, while the remaining one was left. In normal practice, after the testing device filled with culture medium was installed into the incubator, the double-stabilized constant-temperature measurement system was kept closed unless a refill process was needed to change the culture medium. Typical results show that at the beginning of the measurement process, the output readings went through a highly unstable period, although the incubator was set at a constant temperature long before the constant-temperature tent was opened for sample installation. It was due to the high-sensitivity performance of the micro-TFTC sensors which made them quite sensitive to small temperature fluctuations in the measurement system. As the output readings usually started to reach a stable level after a few hours, normally the starting point-in-time selected for the data analysis was not zero-time. For the micro-TFTCs located in different Testing Zones, big or small ones which were filled with culture medium or just empty, their output data show exactly the same up-and-down trend except that their absolute voltage readings were not the same (see Fig. S4 of Supplementary Information). Although the temperature-control system in the incubator provided it with a stability of 0.1 K, due to the differences in thermal conductance and thermal capacitance existing among the measurement instruments, the fluctuation induced by inletting CO_2_ gas for the incubator, and some unknown electrical noise of the whole system, vibrations with a magnitude of 1–2 μV at a long period of time were always observed in the recorded output data. However, the synchronized up-and-down trend of the output data measured from different micro-TFTCs in the same region is a solid evidence that both the measurement equipment and the testing device function well with perfect reliability.

### Weak increments of local temperature in Testing Zones

**Figure 6:**
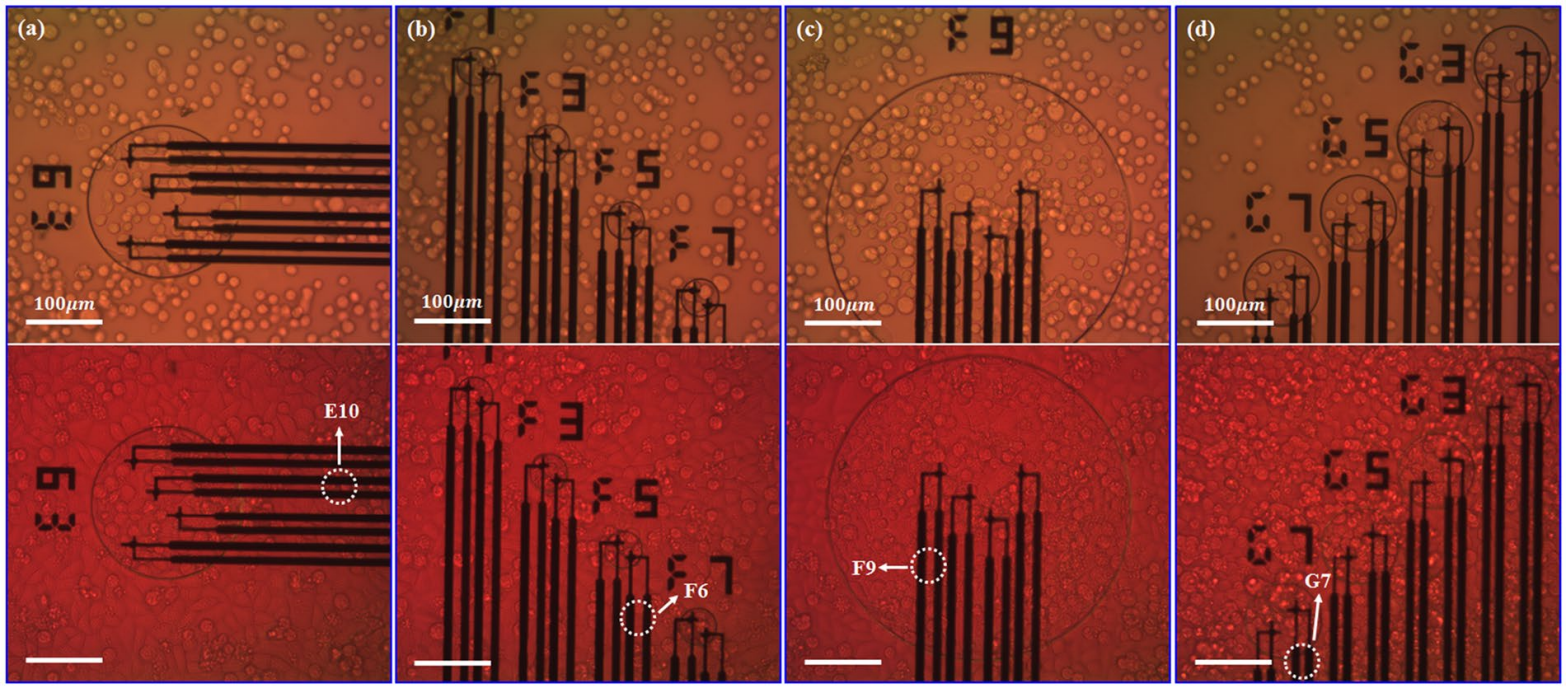
Microscopic images of cultured HepG2 cells. The top panel and bottom panel are photographs taken before and after test, respectively. (**a**–**d**) The relative positions of adherent HepG2 cells and labelled micro-TFTCs E10, F6, F9 and G7. The live cells show typical irregular shape and light contrast, and the apoptotic cells show round shape and dark contrast.

With these special parameters for the facilities, it is able to monitor the increments of local temperature in Testing Zones, which were induced by cellular activities of cultured adherent HepG2 cells. Such an increment occurred a few hours or tens of hours later after the adherent cells were cultured in the incubator. As mentioned in the first paragraph of Results and Discussions, it was reasonable to record increment of local temperatures in a Testing Zone hours later after the culture process started. At the beginning of cell culture process, the TFTC sensors surface at the Testing Zones may not be covered by any cells. When cells grew, they will gradually expanded in number and coverage percentage at the Testing Zones area. Figure 6 shows microscopic images of the HepG2 cells before and after they were cultured in the double-stabilized constant-temperature system for a 57 hours testing. The top panel of Fig. 6(a–d) shows the state of the HepG2 cells when they were just filled into one testing device at four different areas after digestion process. All the cells had spherical appearance with an average diameter of 15–25 μm. The local surface density of the cells was not uniform, spanning from 1800 cells/mm^2^ to 2500 cells/mm^2^, and thus left a large empty space on the device’s surface. For part of the micro-TFTC sensors, each was covered with 1–2 cells. After they were cultured for 57 hours, the device’s surface at the same four areas were all covered by cells shown in the bottom panel of Fig. 6(a–d). Judging from the cellular morphology, there were some apoptotic cells, showing round shape and dark contrast, while the remaining cells seemed to be alive, showing typical irregular shape and light contrast. As the heating power of an individual cell — resulting from its activities of metabolism, reproduction etc. — was very small, only when one or a few adherent cells covered the micro-TFTC surface with close contact as schematically presented in Fig. 4(d), this particular micro-TFTC sensor underneath the cell was capable of sensing the local temperature increment. Figure 4(e) illustrates that by using the designed measurement system with a thermal stability of 10 mK, the increment of local temperature of a live cell firmly stuck to the sensor junction surface in Testing Zone might be detectable. The curve in arbitrary unit indicates that there might be a non-uniform distribution of temperature in a cell.

**Figure 7:**
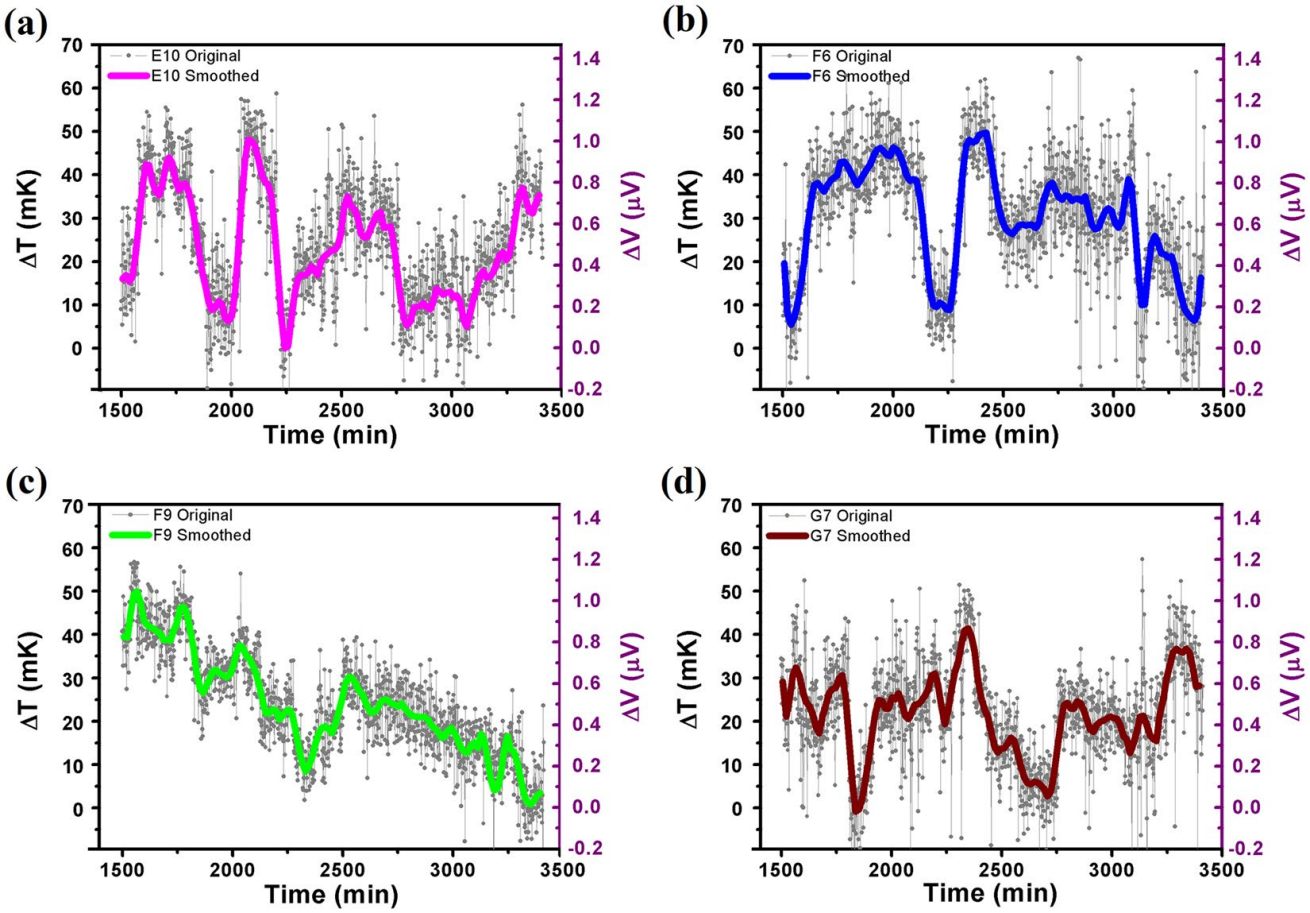
Typical cases of increments of local temperature in Testing Zones recorded with 4 Pd/Cr micro-TFTCs at 32 °C. (**a**–**d**) Measurement data of different Testing Zones taken from Pd/Cr micro-TFTC sensors of E10, F6, F9 and G7, respectively. For the data plotted here, background temperature has been deducted. The grey symbols and lines represent the original data, and the thick lines present the average values of each set of data. Recorded increments of local temperature in Testing Zones are within 60 mK.

Figure 7 presents typical cases recorded with a device sample with a built-in Pd/Cr micro-TFTC array. For this data, the culturing temperature maintained in the incubator was 32 °C. The 4 TFTC sensors, namely E10, F6, F9 and G7, were located in different Testing Zones on the same testing device. The experimental data shown in Fig. 7 are derived from the output readings of the E10, F6, F9 and G7 TFTCs (see Fig. S5 in Supplementary Information) by subtracting the basic background temperature fluctuation measured with a bunch of other TFTCs on the same device (e.g., the TFTCs namely E6, E8, F2, and G5 shown in Fig. S4), which shows synchronized trend as the data of control recorded by TFTCs in Testing Zones without cells, similar to those in Fig. 5(b). Here, the corresponding increments of local temperature in Testing Zone are in the range of 0–60 mK. It is interesting that these four signal curves are different from each other and without a certain periodic.

**Figure 8:**
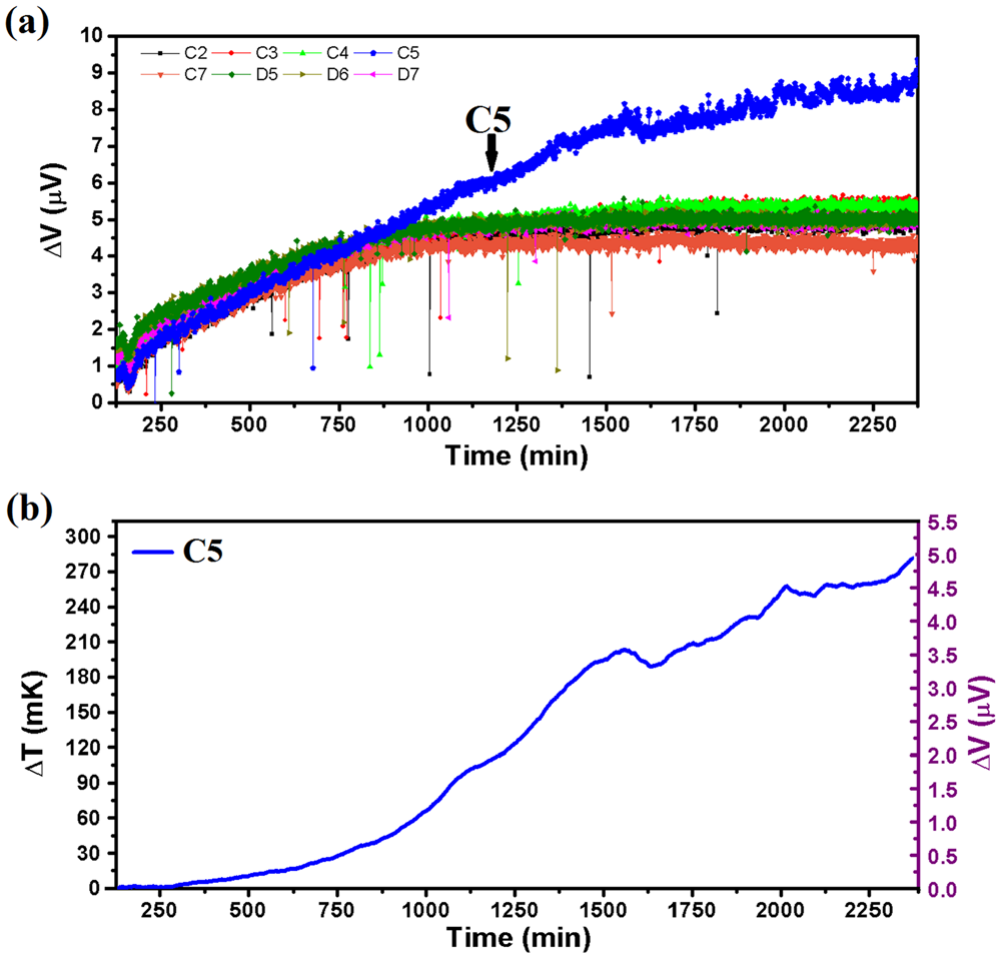
Maximum increment of local temperature in Testing Zone recorded with Cr/Pt micro-TFTCs at 37 °C. (**a**) Output data of 8 Cr/Pt micro-TFTCs (C2, C3, C4, C5, C7, D5, D6 and D7) located in the same region. Except sensor C5 which is rising above, all the other 7 sensors behave according to the same synchronized trend. (**b**) Absolute increment of the output data for sensor C5 from the background temperature. The recorded absolute increment is 285 mK.

Figure 8 presents the largest increment of local temperature in Testing Zone observed in this work, which was recorded at a background temperature of 37 °C. This run of measurement had lasted for about 40 hours without changing culture medium for applying any external stimulus to the cells. As shown in Fig. 8(a), in the first 500 minutes (more than 8 hours), the output readings of the 8 different TFTC sensors located in different Testing Zones (namely C2, C3, C4, C5, C7, D5, D6 and D7) showed similar synchronized trends. Then the output data of sensor C5 gradually increased a bit from the synchronized curves of the rest, and after 500–600 minutes, the trend of measured data of sensor C5 started to rise above the other seven curves. Figure 8(b) plots the subtracted results from the data shown in Fig. 8(a), showing the absolute increments of the output data for sensor C5 from the background temperature. After the adherent HepG2 cells had been cultured for 2200 minutes (around 33 hours), an increment of local temperature in Testing Zone of 285 mK was detected. To show the validity of the recorded data, the same device was cleaned by standard cell-culture procedure to make sure that all cells and residuals were removed from the Testing Zones, and fresh culture medium was added without cells, then the device was put into the incubator for a control run. As shown in Fig. S6 of Supplementary Information, the output data of sensor C5 and the other 7 sensors behaved according to the same synchronized trend. It confirmed that the data shown in Fig. 8(b) were induced by local thermal activities of cultured adherent HepG2 cells rather than by system error or failure of the C5 sensor.

At the current stage, it could be judged from the experimental data that the increment of local temperature in Testing Zone due to cellular activities of target adherent HepG2 cells is pretty weak. The maximum increment of local temperature in Testing Zone of cultured HepG2 cells without additional chemical or physical stimulus was recorded as 285 mK in rare cases, while in most cases it was less than 60 mK. As the temperature recording and morphology analysis were not done at the same time, the direct connection between the recorded increment of local temperature in Testing Zone and certain cell, as well as the status of cell’s life cycle, has not been set up. Efforts for matching the real-time morphology of an individual cell to its real-time 2D mapping of the local temperature is still on-going in our lab. In the improved system, the nano-TFTC sensors^40, 46^ are expected to replace the micro-TFTC sensors used in the present work, so that one single adherent cell could cover more than ten TFTC sensors, leading to a 2D map of the local temperature distribution. By comparing this 2D map with the remaining empty surface region without cells, it is possible to confirm the origin of the increment in temperature. Furthermore, by comparing the real-time microscopic image of the target cell sitting on the nano-TFTC array, it is possible to determine the correlation between its local temperature history and its life cycle, either in its natural state or under different external stimulus.

## Conclusion

In this work, we precisely measured the local temperature increment induced by cultured adherent HepG2 cells. The cells were cultured in an environment that had been maintained at a constant temperature with a uniquely designed system, which consisted of a commercial constant-temperature incubator for cell culture, and a controllable constant-temperature tent to hold the incubator. In particular, all the measurement instruments, including the testing device, the multiplexer and the nanovoltmeter were put into the incubator to keep the same constant temperature and avoid extra thermal and electrical noises. By using these extreme measures and high-performance Pd/Cr or Cr/Pt micro-TFTC array devices, a thermal stability of 10 mK for the system was achieved. The local temperatures in Testing Zones where HepG2 cells were cultured directly on top of the micro-TFTC array in culture medium were continuously monitored for up to 57 hours, which covered several life cycles of the cells under test. Statistics analysis of dozens of runs of experiments showed that, the increment of local temperature was within 60 mK in most cases in Testing Zones where adherent cells might stick firmly to micro-TFTC sensors. In rare cases, larger increment of local temperature in Testing Zone up to 285 mK was detected. The method presented in this work has set up solid foundation for the realization of real-time, 2D mapping of the precise local temperature distribution of individual live cells with a nano-sized thermocouple array.

## Methods

### Double-stabilized measurement system

Photographs of the cellular temperature measurement system are presented in Supplementary Information (see Fig. S3). As schematically illustrated in Fig. 4(a–c), a commercial constant-temperature incubator (BPH -9272, Shanghai Yiheng, CN) with a stability of 0.1 K was located in a homemade, constant-temperature tent with a volume of 2.0 × 2.0 × 1.65 m^3^. This constant-temperature tent was constructed with an aluminum alloy frame, two layers of thermally insulating glass fiber cloth with aluminum foil coating, heating wires and other thermal insolating materials. Three temperature controllers (TC-05B, Sieval Corp., CN) were used to maintain a constant temperature of the tent, with a stability of 0.5 K. The measurement instruments including a device with build-in micro-TFTC array, a 10 × 10 multiplexer^47^, a nanovoltmeter (Keithley 2182 A) and connecting cables were put into the incubator to keep them at the same constant temperature. Data acquisition was controlled by a computer located on the outside of the constant-temperature tent with NI interface cards and LabVIEW programs.

### Device fabrication and calibration

Testing devices with Pd/Cr (or Cr/Pt) micro-TFTC array were fabricated on 2 inch diameter glass substrates (Jingjiguangxue, CN) with standard cleanroom techniques for semiconductor micro-electronics. Lithography of the micro-TFTC patterns (see Fig. 1(a)) was completed by a MJB4 mask aligner (SUSS MicroTec, GER) operating with 350 nm ultraviolet light. Pd thin films were deposited with an electron-stripe evaporator (DE400, DE Tec, CN), and Cr, Pt thin films were deposited with a magnetron sputtering system (PVD75, Kurt J. Lesker, USA) operating in Ar atmosphere. Before film deposition, the residual photoresist on a patterned substrate was removed by oxygen plasma at a power of 250 W for 30 s in a plasma generator (PDC-M, PVA TePla, GER). The 20 μm deep Testing Zones (see Fig. 1(b)) were defined directly by a layer of SU-8 photoresist (No. 3025, MicroChem, USA) with lithography process.

In order to further reduce electrical noises, a 2-nm thick HfO_2_ insulating layer was deposited on top of the as-fabricated micro-TFTC array with atomic-layer-deposition technique (Savannah S100, Cambridge NanoTech, UK). Leaking testing among neighboring TFTC sensors in the same Testing Zone was performed with a semiconductor characterization system (Keithley 4200-SCS) under varied surrounding media of air, culture medium and HCl aqueous solutions with pH values of 2, 4 and 6, respectively.

On top of the Testing Zones, a 10 mm thick polydimethylsiloxane (PDMS) layer, was covered to leave 4 cylindroid open rooms for cell culture (Fig. 1(c)). This PDMS layer, consisting pre-polymer (base) and cross-linker (curing agent) components of PDMS (Sylgard 184, Dow Corning Corp), was made with a plastic mold fabricated by 3D printing technique. The pre-polymer base and cross-linker curing agent were mixed at a volume ratio of 10:1 at room temperature^48^, then were poured into the mold and baked at 70 °C for one hour.

Micro-TFTC samples were calibrated on a homemade calibration platform. For calibration, long TFTC samples were fabricated on 4-inch diameter glass substrates under the same fabrication conditions as the micro-TFTCs used for the testing devices for cellular temperature measurements. The stripe width for the calibration samples spanned from 20 μm to 2000 μm, while the stripe length was kept at 80 mm or 100 mm. During calibration, the cold-ends of the samples were kept at a stable temperature of 16 °C, and the temperature of the hot-ends was increased from 50 °C to 150 °C. Measurements with a Keithley 2182 A nanovoltmeter and a computerized data acquisition system^47^ were performed after the temperatures at both ends were stabilized, as indicated by commercial K-type thermocouples.

### Cell culture process

Human hepatoblastoma (HepG2) cells used in this work were derived from hepatocellar carcinoma of human. They were cultured in 25 ml culture flasks (Corning, NY, USA) with RPMI1640 medium (Corning, NY, USA) at 37 °C in an incubator in 5% CO_2_ mixture atmosphere. The medium used here was supplemented with 10% (v/v) fetal bovine serum (FBS) (Sijiqing, China) and 1% penicillin (Corning, NY, USA). The HepG2 cells were passaged at a split ration of 1:3 every week and the medium was changed every 2–3 days. For experiment, HepG2 cells were seeded in a 1 cm diameter tube at a density around 5 × 10^5^ mL^−1^.

## Electronic supplementary material

Supplementary Information

## References

[1] Paulik MA (1998). Development of infrared imaging to measure thermogenesis in cell culture: thermogenic effects of uncoupling protein-2, troglitazone, and beta-adrenoceptor agonists. Pharmaceutical Research..

[2] Zohar O (1998). Thermal imaging of receptor-activated heat production in single cells. Biophysical Journal..

[3] Sakaguchi R, Kiyonaka S, Mori Y (2015). Fluorescent sensors reveal subcellular thermal changes. Current Opinion in Biotechnology..

[4] Lowell BB, Spiegelman BM (2000). Towards a molecular understanding of adaptive thermogenesis. Nature..

[5] Vetrone F (2010). Temperature sensing using fluorescent nanothermometers. Acs Nano..

[6] Monti M, Brandt L, Ikomi-Kumm J, Olsson H (1986). Microcalorimetric investigation of cell metabolism in tumour cells from patients with non-Hodgkin lymphoma (NHL). Scandinavian Journal of Haematology..

[7] Kuruganti PT, Qi H (2002). Asymmetry analysis in breast cancer detection using thermal infrared images. Proceedings of the Second Joint EMBS/BMES Conference..

[8] Mccabe KM, Hernandez M (2010). Molecular Thermometry. Pediatric Research..

[9] Brites CD (2012). Thermometry at the nanoscale. Nanoscale..

[10] Jaque D (2014). Fluorescent nanothermometers for intracellular thermal sensing. Nanomedicine..

[11] Bai T, Ning G (2016). Micro/Nanoscale Thermometry for Cellular Thermal Sensing. Small..

[12] Binslem SA, Ahmad MR, Awang Z (2015). Intracellular Thermal Sensor for Single Cell Analysis - Short review. Jurnal Teknologi (Sciences & Engineering)..

[13] Jaque D, Vetrone F (2012). Luminescence nanothermometry. Nanoscale..

[14] Walker GW (2003). Quantum-dot optical temperature probes. Applied Physics Letters..

[15] Gota C, Okabe K, Funatsu T, Harada Y, Uchiyama S (2009). Hydrophilic Fluorescent Nanogel Thermometer for Intracellular Thermometry. Journal of the American Chemical Society..

[16] Benayas A, Escuder E, Jaque D (2012). High-resolution confocal fluorescence thermal imaging of tightly pumped microchip Nd:YAG laser ceramics. Applied Physics B..

[17] Okabe K (2012). Intracellular temperature mapping with a fluorescent polymeric thermometer and fluorescence lifetime imaging microscopy. Nature Communications..

[18] Baffou G, Kreuzer MP, Kulzer F, Quidant R (2009). Temperature mapping near plasmonic nanostructures using fluorescence polarization anisotropy. Optics Express..

[19] Arai S (2015). Micro-thermography in millimeter-scale animals by using orally-dosed fluorescent nanoparticle thermosensors. Analyst..

[20] Kucsko G (2013). Nanometre-scale thermometry in a living cell. Nature..

[21] Yang JM, Yang H, Lin L (2011). Quantum dot nano thermometers reveal heterogeneous local thermogenesis in living cells. Acs Nano..

[22] Tanimoto R (2016). Detection of Temperature Difference in Neuronal Cells. Scientific Reports..

[23] Tseeb V, Suzuki M, Oyama K, Iwai K, Ishiwata SI (2009). Highly thermosensitive Ca^2+^ dynamics in a HeLa cell through IP3 receptors. Hfsp Journal..

[24] Hayashi T, Fukuda N, Uchiyama S, Inada N (2015). A cell-permeable fluorescent polymeric thermometer for intracellular temperature mapping in mammalian cell lines. Plos One..

[25] Kiyonaka S (2013). Genetically encoded fluorescent thermosensors visualize subcellular thermoregulation in living cells. Nature Methods..

[26] Donner J, Thompson SA, Kreuzer MP, Baffou G, Quidant R (2012). Mapping intracellular temperature using Green Fluorescent Protein - From *in vitro* to *in vivo*. Nano Letters..

[27] Itoh H (2016). Direct organelle thermometry with fluorescence lifetime imaging microscopy in single myotubes. Chemical Communications..

[28] Arai S (2015). Mitochondria-targeted fluorescent thermometer monitors intracellular temperature gradient. Chemical Communications..

[29] Arai S, Lee SC, Zhai D, Suzuki M, Chang YT (2014). A molecular fluorescent probe for targeted visualization of temperature at the endoplasmic reticulum. Scientific Reports..

[30] Reschgenger U, Grabolle M, Cavalierejaricot S, Nitschke R, Nann T (2008). Quantum dots versus organic dyes as fluorescent labels. Nature Methods..

[31] Shang L, Stockmar F, Azadfar N (2013). Intracellular Thermometry by Using Fluorescent Gold Nanoclusters^†^. Angewandte Chemie International Edition..

[32] Albers AE (2012). Dual-emitting quantum dot/quantum rod-based nanothermometers with enhanced response and sensitivity in live cells. Journal of the American Chemical Society..

[33] Uchiyama S (2015). A cationic fluorescent polymeric thermometer for the ratiometric sensing of intracellular temperature. Analyst..

[34] Baffou G, Rigneault H, Marguet D, Jullien L (2014). A critique of methods for temperature imaging in single cells. Nature Methods..

[35] Baffou G, Rigneault H, Marguet D, Jullien L (2015). The 10(5) gap issue between calculation and measurement in single-cell thermometry Reply. Nature Methods..

[36] Suzuki M, Zeeb V, Arai S, Oyama K, Ishiwata S (2015). The 10(5) gap issue between calculation and measurement in single-cell thermometry. Nature Methods..

[37] Kiyonaka S (2015). Validating subcellular thermal changes revealed by fluorescent thermosensors. Nature Methods..

[38] Suzuki M, Tseeb V, Oyama K, Ishiwata S (2007). Microscopic Detection of Thermogenesis in a Single HeLa Cell. Biophysical Journal..

[39] Jang M (2009). The Characteristics of Seebeck Coefficient in Silicon Nanowires Manufactured by CMOS Compatible Process. Nanoscale Research Letters..

[40] Huo X, Wang Z, Fu M, Xia J, Xu S (2016). A sub-200 nanometer wide 3D stacking thin-film temperature sensor. Rsc Advances..

[41] Lee W (2013). Heat dissipation in atomic-scale junctions. Nature..

[42] Watanabe, M. S., Kakuta, N., Mabuchi, K. & Yamada, Y. Micro-thermocouple probe for measurement of cellular thermal responses, *IEEE Engineering in Medicine & Biology 27th Annual Society Conference*. 4858–4861 (2005). 10.1109/IEMBS.2005.1615560 17281330

[43] Wang C (2011). Determining intracellular temperature at single-cell level by a novel thermocouple method. Cell Research..

[44] Tian W (2015). A high precision apparatus for intracellular thermal response at single-cell level. Nanotechnology..

[45] Liu H, Sun W, Chen Q, Xu S (2011). Thin-Film Thermocouple Array for Time-Resolved Local Temperature Mapping. IEEE Electron Device Letters..

[46] Huo X, Xu J, Wang Z, Yang F, Xu S (2016). Performance of Nano-Submicron-Stripe Pd Thin-Film Temperature Sensors. Nanoscale Research Letters..

[47] Li G (2016). Real-Time Two-Dimensional Mapping of Relative Local Surface Temperatures with a Thin-Film Sensor Array. Sensors..

[48] Mata A, Fleischman AJ, Roy S (2006). Characterization of polydimethylsiloxane (PDMS) properties for biomedical micro/nanosystems. Biomedical Microdevices..

