# Supplementary material for: Measurement of local temperature increments induced by cultured HepG2 cells with micro-thermocouples in a thermally stabilized system
Source: Sci Rep. 2017 May 11;7:1721. doi: 10.1038/s41598-017-01891-1 (PMC5431931; doi:10.1038/s41598-017-01891-1)
Supplement: Supplementary file 1 — Supplementary Information [file 41598_2017_1891_MOESM1_ESM.pdf]

# Measurement of local temperature increments induced by cultured HepG2 cells with micro-thermocouples in a thermally stabilized system

**Fan Yang[1], Gang Li[1], Jiamin Yang[2], Zhenhai Wang[1], Danhong Han[1], Fengjie Zheng[2] & Shengyong Xu[1*]**

[1] Key Laboratory for the Physics & Chemistry of Nanodevices, and Department of Electronics, Peking University, Beijing, 100871, P. R. China

[2] School of Basic Medical Sciences, Beijing University of Chinese Medicine, Beijing 100871, P. R. China

Correspondence author: xusy@pku.edu.cn

**Contents**

**Supplementary Table S1**: Calibration data of Pd/Cr and Cr/Pt TFTCs with different stripe width and length................................................ 2

**Supplementary Figure S1**: Photographs of fabrication processes for the device with build-in micro-TFTC array......................................... 3

**Supplementary Figure S2:** Measurement data for control experiments tested with aqueous solutions of different pH values and $Ca^{2+}$ concentrations............................................................. 4

**Supplementary Figure S3:** Overall distribution of cellular temperature measurement system......................................................................... 5

**Supplementary Figure S4**: Thermal fluctuations of Cr/Pt micro-TFTC array tested in the cellular measurement system at 37℃...................... 6

**Supplementary Figure S5**: Output data of 8 Pd/Cr micro-TFTCs tested in the cellular temperature measurement at 32℃................................ 7

**Supplementary Figure S6:** Thermal fluctuations of 8 Cr/Pt micro-TFTCs tested in a control experiment at 37℃............................................ 8

**Supplementary Figure S7:** Contact modes between adherent HepG2 cell and substrate surface......................................................................... 9

**Supplementary Figure S8:** Relative locations between adherent cell and micro-TFTC sensor......................................................................... 10

**Supplementary Table S1. Calibration data of Pd/Cr and Cr/Pt TFTCs with different stripe width and length.**

| NO. | Stripe width (μm) | Stripe length (mm) | Thermopower of Pd/Cr TFTCs (μV/K) | Thermopower of Cr/Pt TFTCs (μV/K) |
|---|---|---|---|---|
| 1 | 2 | 80 | 20.83 | 17.51 |
| 2 | 20 | 80 | 20.83 | 17.50 |
| 3 | 20 | 100 | 21.00 | 17.55 |
| 4 | 50 | 80 | 20.76 | 17.69 |
| 5 | 100 | 80 | 20.95 | 17.62 |
| 6 | 200 | 80 | 21.03 | 17.62 |
| 7 | 500 | 80 | 21.12 | 17.60 |
| 8 | 1000 | 80 | 21.13 | 17.59 |
| 9 | 2000 | 80 | 21.29 | 17.65 |

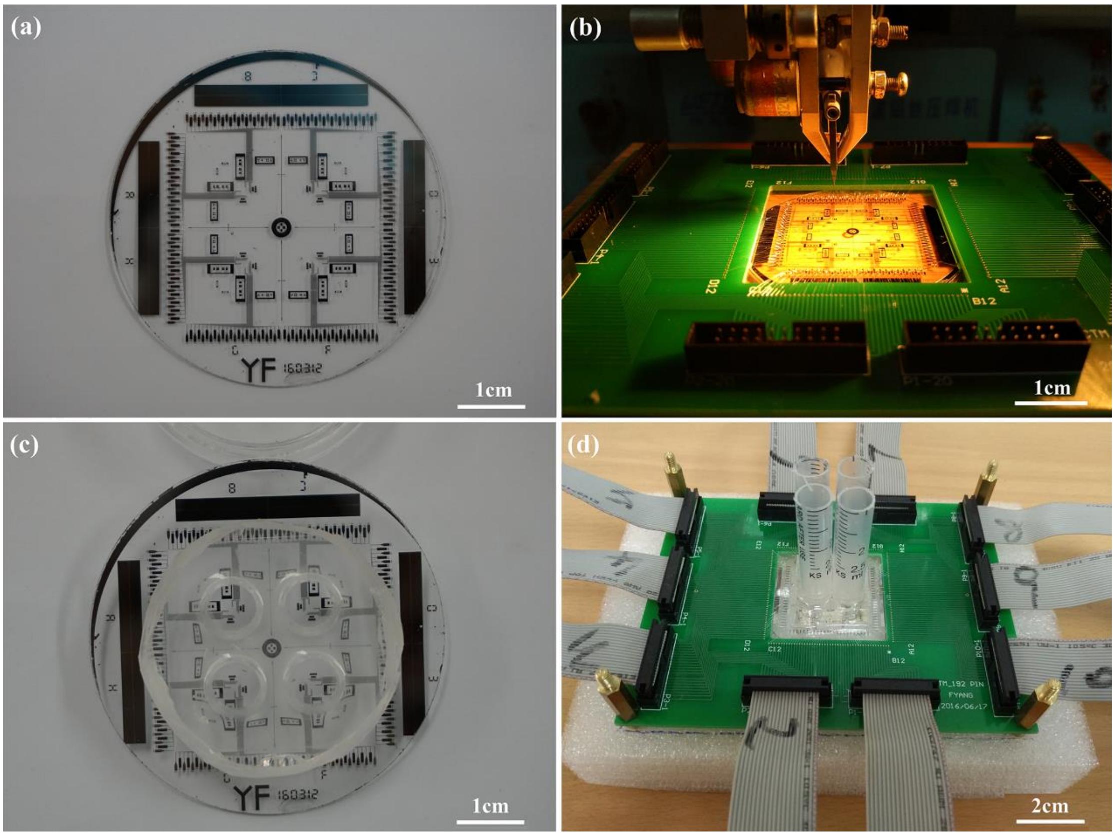


**Supplementary Figure S1: Photographs of fabrication processes for the device with build-in micro-TFTC array.** (**a**) A Pd/Cr micro-TFTC array was prepared on the glass substrate. (**b**) The micro-TFTC array sample was bonded to the Printed Circuit Board through the wire bonding process. (**c**) A piece of PDMS sample with cylindroid rooms. (**d**) A measurable device with build-in Pd/Cr micro-TFTC array.

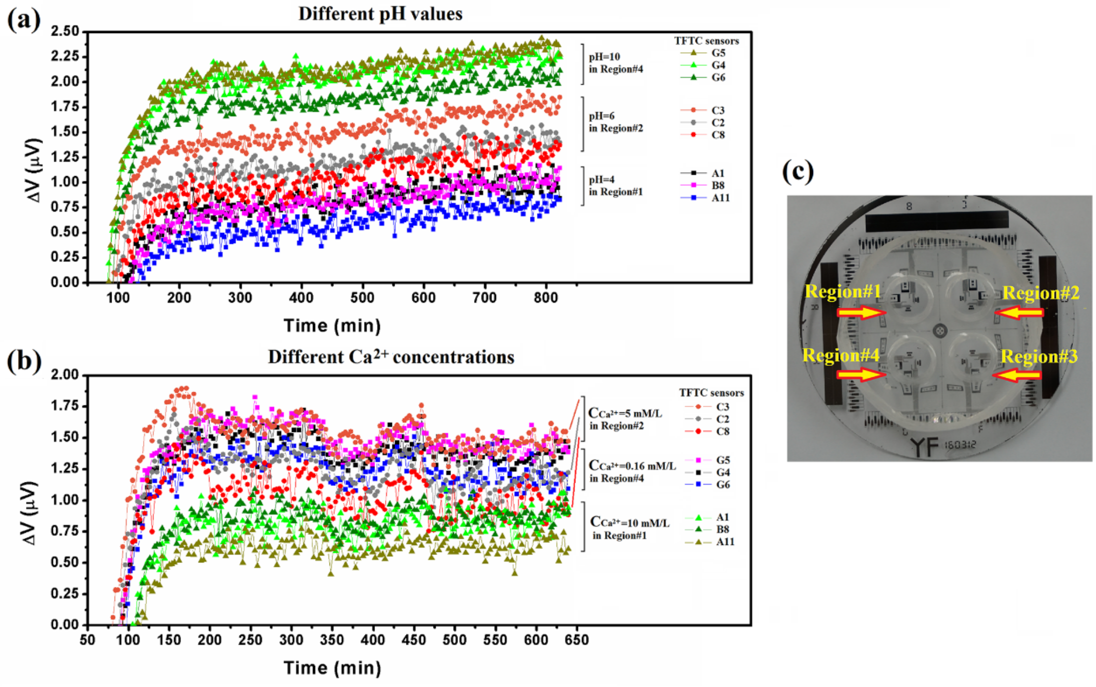


**Supplementary Figure S2: Measurement data for control experiments tested with aqueous solutions of different pH values and $Ca^{2+}$ concentrations. (a)** Continuous measurement data of over 10 hours for Pd/Cr micro-TFTCs under solutions with pH values of 4, 6 and 10 for the Regions #1, #2 and #4 shown in **(c)**. **(b)** In another run, data of continuous measurement over 10 hours for the same device under $CaCl_2$ solutions with varied $Ca^{2+}$ concentrations of 0.16 mM/L, 5.0 mM/L and 10.0 mM/L for the Regions #4, #2 and #1, respectively. The same trend of fluctuations of the output readings in (a) and (b) shows that the micro-TFTCs are insensitive with the variations in the pH value and ion concentration of the culture medium.

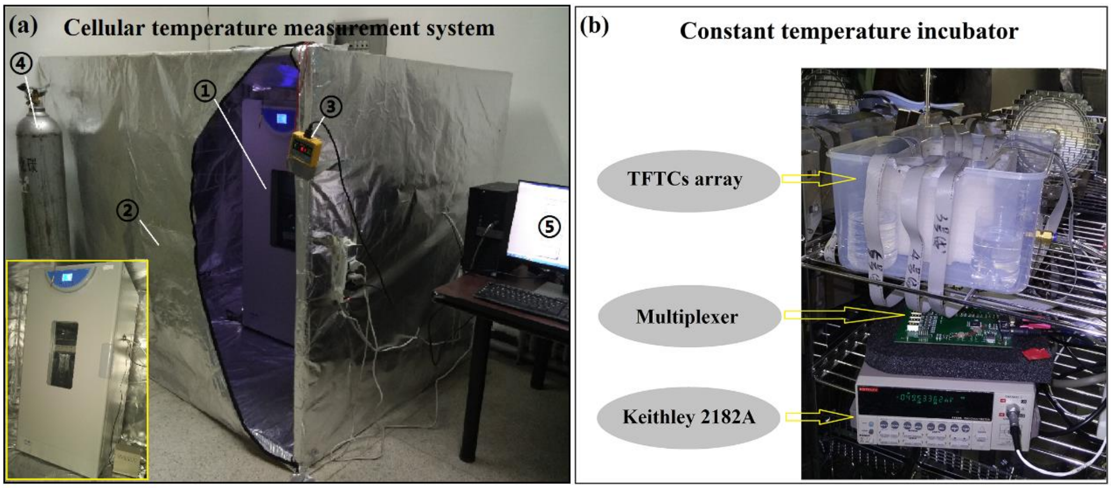


**Supplementary Figure S3: Overall distribution of cellular temperature measurement system.** (**a**) Photograph of the cellular temperature measurement system. ① is the commercial constant-temperature incubator and its appearance is shown in the inset, ② is the constant- temperature tent, ③ is the temperature controller, ④ is the $CO_2$ Gas cylinder and ⑤ is the computerized data acquisition system. (**b**) Internal distribution of the commercial constant-temperature incubator. The device with build-in micro-TFTC array is on the top layer, the 10×10 multiplexer is in the middle position, and the Keithley 2182A nanovoltmeter is on the bottom layer.

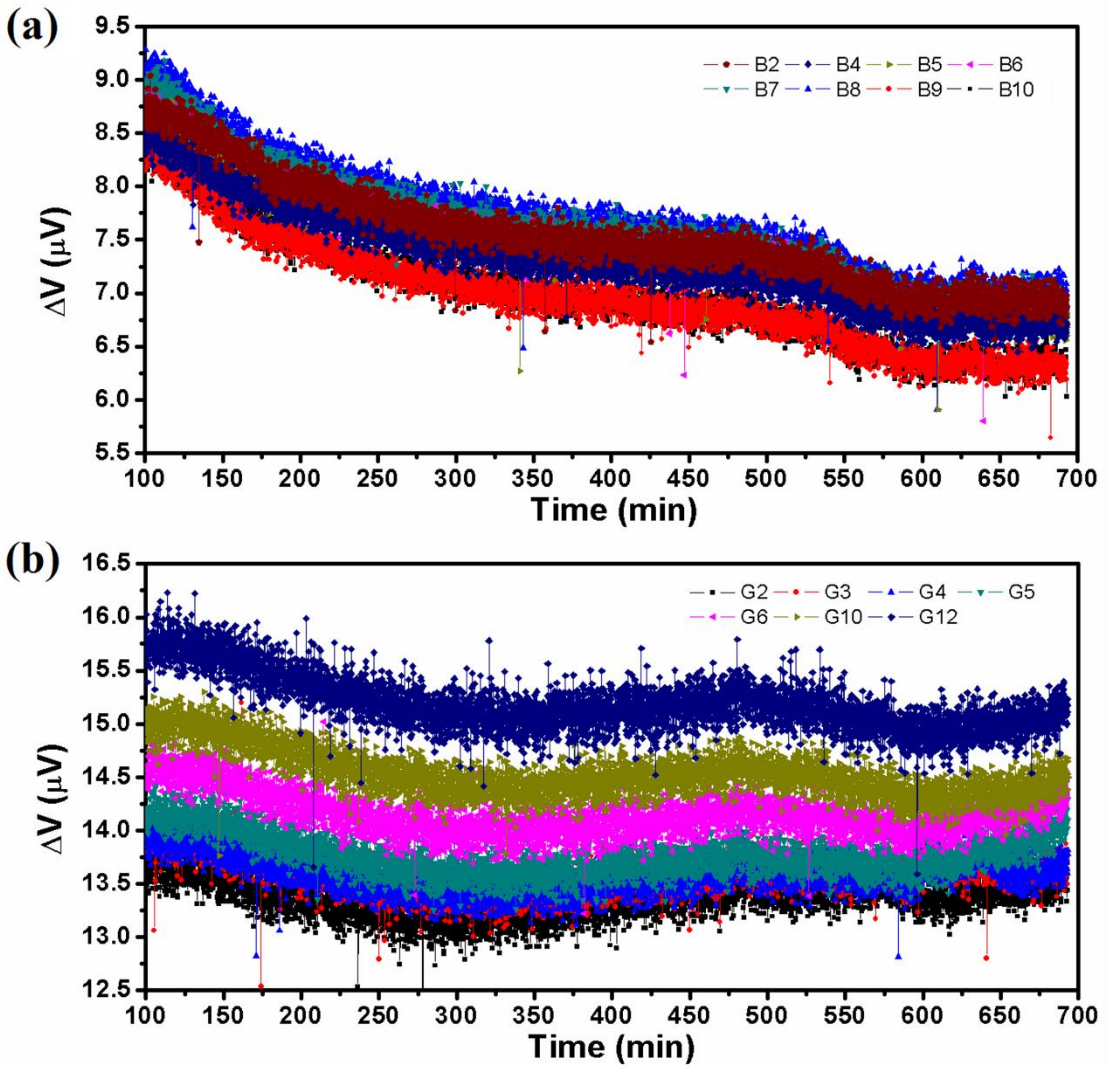


**Supplementary Figure S4: Thermal fluctuations of Cr/Pt micro-TFTC array device tested in the cellular measurement system at 37℃.** (**a**) Thermal fluctuations of 8 Cr/Pt TFTCs (B2, B4, B5, B6, B7, B8, B9 and B10 located in the same region), tested exposed to the air. (**b**) Thermal fluctuations of the other 7 Cr/Pt TFTCs (G2, G3, G4, G5, G6, G10 and G12 located in the same region) tested in a liquid environment.

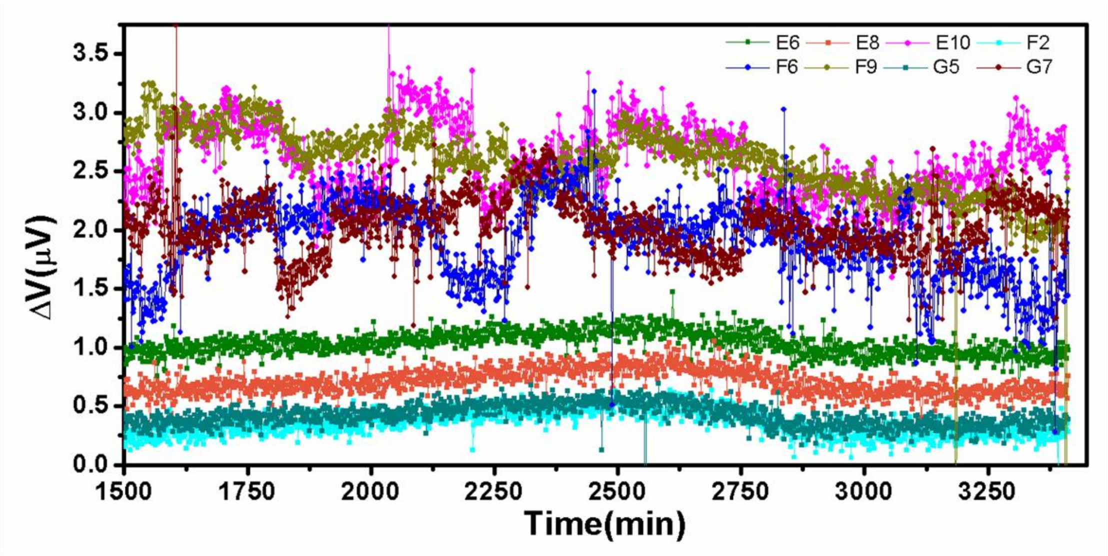


**Supplementary Figure S5: Output data of 8 Pd/Cr micro-TFTCs tested in the cellular temperature measurement at 32℃.** Among these 8 Pd/Cr micro-TFTCs, E6, E8, E10, F2, F6 and F9 are located in the same region, G5 and G7 are located in another region. The 4 TFTCs E6, E8, F2, and G5 show synchronized trend which represents the fluctuations of backgroud temperature.While the other 4 TFTCs E10, F6, F9 and G7 show different trends of fluctuations.

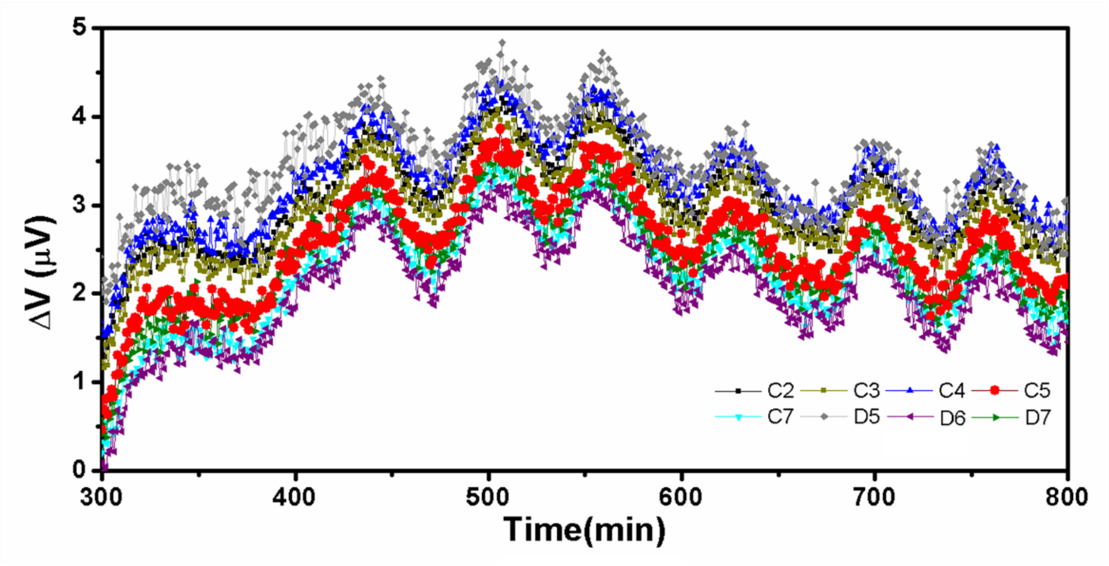


**Supplementary Figure S6: Thermal fluctuations of 8 Cr/Pt micro-TFTCs tested in a control experiment at 37℃.** These Cr/Pt TFTCs (C2, C3, C4, C5, C7, D5, D6 and D7) are located in the same region, but different Testing Zones. The red symbols and line show the trend of TFTC C5. It has a synchronized trend with the other 7 TFTCs.

Because to the best of our knowledge, no report describes the details on how the adherent HepG2 cell contacts to the glass substrate surface. There could be many different configurations. We provide four kinds of the contact modes between adherent HepG2 cell and substrate surface here.

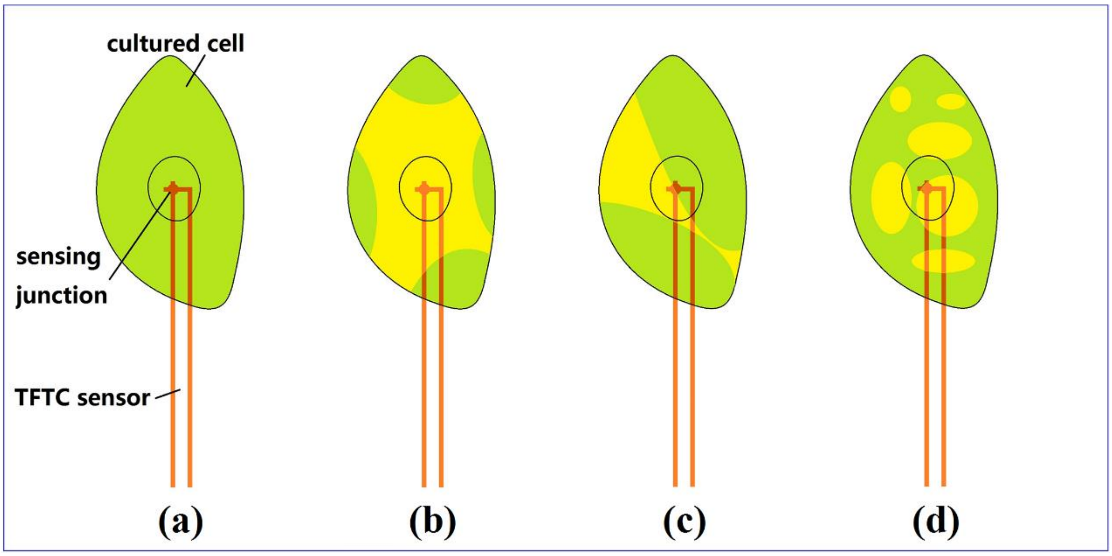


**Supplementary Figure S7: Contact modes between adherent HepG2 cell and substrate surface.** The contact regions are highlighted with green color, the gaps filled with culture medium are represented with yellow color. **(a)** Cell's bottom membrane fully covers the substrate surface. Under this mode, the particular micro-TFTC may detect the temperature fluctuation induced by cell. **(b)** Some edges of cell's membrane contact to the substrate surface, leaving a big gap between the cell membrane and the sensing area of micro-TFTC filled with culture medium. Under this mode, the particular micro-TFTC can detect the ambient temperature fluctuation in the culture medium. **(c) & (d)** Cell's bottom membrane covers the substrate surface partially. Under these modes, the particular micro-TFTC may give out the temperature fluctuation induced by cell or the ambient temperature fluctuation in the culture medium. It all depends.

For the better understanding of the recorded increments of local temperature in Testing Zones induced by cultured cells, we also provide different relative locations between adherent cell and micro-TFTC sensor as Fig. S8 here. Although the HepG2 cell is one kind of adherent cells, it doesn't keep stationary at a certain position, and may first moves onto a micro-TFTC sensor then moves away due to the cells' extrusion, cell division, or other reasons, thus change the relative locations to the micro-TFTC sensor. Combining the relative locations between adherent cell and micro-TFTC sensor the contact modes between adherent HepG2 cell and substrate surface, one will know that there could be many different shapes in the recorded data.

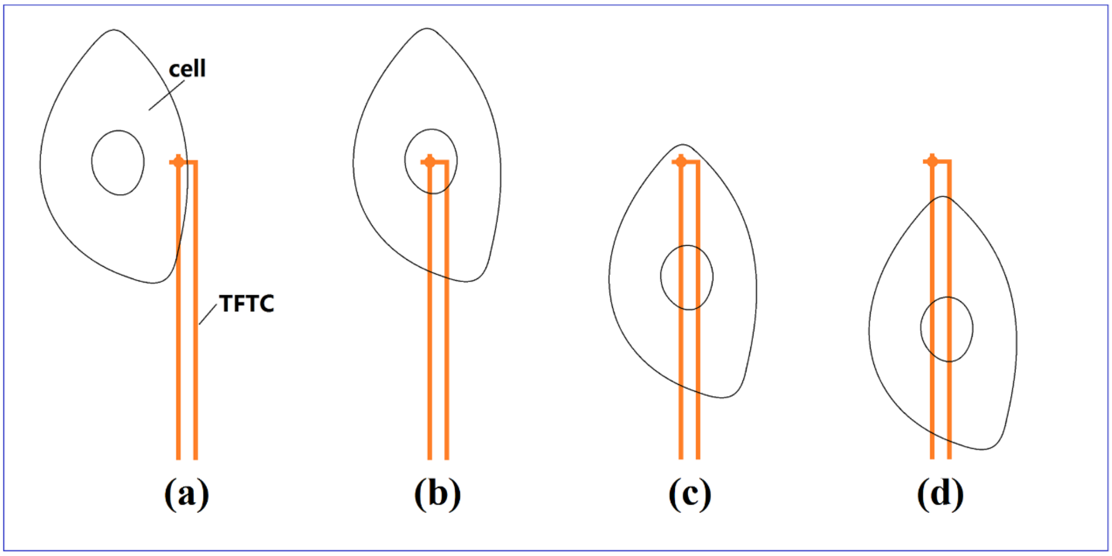


**Supplementary Figure S8: Relative locations between adherent cell and micro-TFTC sensor. (a)** The cell is approaching to the micro-TFTC sensor with a small part of covering. **(b)** The cell is on the top of the micro-TFTC sensor with fully covering. **(c)** The cell is moving away from the micro-TFTC sensor leaving a small part of covering. **(d)** The cell is completely moving out of the micro-TFTC sensor's sensing area.
